# Foster Care Leads to Lower Irritability Among Adolescents with a History of Early Psychosocial Deprivation

**DOI:** 10.1007/s10802-024-01193-x

**Published:** 2024-04-20

**Authors:** Yanbin Niu, George A. Buzzell, Ana Cosmoiu, Nathan A. Fox, Charles A. Nelson, Charles H. Zeanah, Kathryn L. Humphreys

**Affiliations:** 1 Department of Psychology & Human Development, Peabody College, Vanderbilt University, 230 Appleton Place, Nashville, TN 37203 USA; 2 Department of Psychology, Florida International University, Miami, FL USA; 3 Department of Psychology and Cognitive Sciences, University of Bucharest, Bucharest, Romania; 4 Department of Human Development and Quantitative Methodology, University of Maryland, College Park, MD USA; 5 Boston Children’s Hospital of Harvard Medical School, Boston, MA USA; 6 Harvard Graduate School of Education, Cambridge, MA USA; 7 Department of Psychiatry and Behavioral Sciences, Tulane University School of Medicine, New Orleans, LA USA

**Keywords:** Irritability, Early caregiving environment, Institutional care, Adolescence

## Abstract

Irritability reflects a propensity for frustration and anger, and is a transdiagnostic symptom of both externalizing and internalizing psychopathology. While early adverse experiences are associated with higher levels of irritability, experiences of early psychosocial deprivation and whether family-based placements can mitigate the impact on subsequent irritability, remain underexplored. The current study examined irritability in 107 16-year-olds with a history of institutional care from a randomized controlled trial of foster care as an alternative to institutional care and 49 community comparison children. At age 16 years, irritability was assessed using parent- and self-report forms of the Affective Reactivity Index. Compared to community adolescents, those with a history of institutional care exhibited significantly elevated irritability levels. Among those who experienced institutional care, those randomized to foster care had lower levels of irritability compared to participants randomized to the care-as-usual group, and this effect persists after controlling for baseline negative emotionality. These findings suggest a causal link between high-quality foster care and lower irritability following psychosocial deprivation. Additionally, longer duration in institutional care and non-family placement at age 16 years were associated with higher levels of irritability, highlighting the role of caregiving in explaining variation in irritability in adolescence. Policies that support long-term, high-quality family placements for children without regular caregivers should be prioritized.

## Introduction

Irritability reflects a propensity for frustration and anger (Leibenluft & Stoddard, 2013; Vidal-Ribas et al., 2016). Children with heightened irritability tend to have a lower threshold for anger, resulting in more frequent and intense temper outbursts compared to their peers (Leibenluft & Stoddard, 2013; Vidal-Ribas et al., 2016). These outbursts often involve physical and verbal displays of anger, and between outbursts, irritable children may also experience a persistent angry mood, including sulky behaviors and a feeling of annoyance over extended periods of time (Berkowitz, 1989; Brotman et al., 2017). Additionally, irritability is transdiagnostic, with links to both concurrent and prospective symptoms of multiple forms of externalizing and internalizing psychopathology (Brotman et al., 2006; Copeland et al., 2015; Dougherty et al., 2013; Humphreys et al., 2019; Leibenluft et al., 2006; Pickles et al., 2010; Stringaris et al., 2009, 2013; Stringaris & Goodman, 2009). For instance, irritability is represented in more than a dozen psychiatric disorders in the 5th Edition of the Diagnostic and Statistical Manual of Mental Disorders (DSM-5; American Psychiatric Association, 2013). As a public health concern, irritability is associated with suicidal risk in children and adolescents (i.e., suicidal ideation and suicide attempts) (Benarous et al., 2019; Conner et al., 2004; Liu et al., 2021; Orri et al., 2018, 2019; Pickles et al., 2010). Furthermore, increased irritability is linked to diverse adverse outcomes, such as impairment in family and peer relationships (Chad-Friedman et al., 2022; Dougherty et al., 2013), difficulties in academics and self-esteem (Leppert et al., 2019), school suspensions (Copeland et al., 2015), lower work productivity (Jha et al., 2021), and global functioning (Chad-Friedman et al., 2022; Kircanski et al., 2017).

Given the broad and profound implications associated with irritability, it is important to investigate potential risk factors that may contribute to its development. While a portion of variability in irritability can be attributed to genetics, there is evidence that the environment also influences irritability. For example, early adverse experiences are associated with greater irritability (Bielas et al., 2016; Oliver, 2015; Pagliaccio et al., 2018; Ravi et al., 2022; Yu et al., 2023). Further, cumulative adverse childhood experiences were found to be associated with higher levels of irritability in a sample of adolescent boys (Bielas et al., 2016). Another longitudinal study identified 3 latent classes of irritability trajectory from preschool through late childhood/early adolescence, and children with a “high irritability” trajectory—characterized by heightened baseline irritability that remained elevated across development—had more adverse experiences at baseline (Pagliaccio et al., 2018). Moreover, in a large, national dataset with over 4,000 children, composite parenting score, comprised of physical assault, psychological abuse, and neglect, was associated with elevated levels of irritability at one or more points during childhood and adolescence, as compared to the group characterized by overall low irritability (Yu et al., 2023). Thus, data from these studies suggest the possibility that adolescents exposed to early adversity, including the type of psychosocial neglect experienced in institutional care, may exhibit higher levels of irritability.

In contrast to the potential for adverse experiences to increase the risk for irritability, positive caregiving experiences may prevent or mitigate risks for heightened irritability. For instance, a joint parent–child intervention aimed at addressing anger and irritability in children diagnosed with severe mood dysregulation (e.g., assisting with emotion regulation, and appropriately responding to oppositional behaviors) reduced child irritability (Waxmonsky et al., 2016). Additionally, a randomized trial of the Family Check-Up (FCU) program in early childhood was designed to improve children’s adjustment through family management practices, including positive behavior support, monitoring and limit setting, and family relationship building. The program resulted in lower irritability in four-year-olds, which in turn predicted lower externalizing and internalizing symptoms at age 10.5 years (Smith et al., 2019). While parenting interventions have demonstrated changes in child irritability, it remains unclear whether improved caregiving can reduce the risk of irritability following exposure to severe adversity and its long-term efficacy.

In the present study, we examined irritability at age 16 years using data from the Bucharest Early Intervention Project (BEIP) (Zeanah et al., 2003), a randomized controlled trial (RCT) of foster care as an alternative to institutional care for infants and young children residing in psychosocially depriving institutions in Bucharest, Romania. Children were randomized to one of two groups: (1) high-quality foster care (Foster Care Group; FCG), or (2) care as usual (Care as Usual Group; CAUG), which typically resulted in more prolonged institutional care exposure. A group of age- and gender-matched children who had never experienced institutional rearing was recruited from the community in order to serve as a comparison group (never-institutionalized group; NIG). We hypothesized that: (1) children with a history of institutional care would exhibit higher levels of irritability compared to those never institutionalized; (2) children in FCG would have lower levels of irritability compared to those in CAUG. Furthermore, we investigated the associations between irritability and factors that may explain the variability of potential beneficial effects of foster care, such as the percentage of time spent in institutional care, age of placement into foster care, and current placement at the time of the assessment. Consistent with prior studies (Buzzell et al., 2020; Humphreys et al., 2022; McDermott et al., 2013; Troller-Renfree et al., 2016), we hypothesized that lower levels of irritability would be associated with a short percent time in institutional care, a younger age of placement into foster care, and current residence with a family compared to those not residing with a family.

## Method

### Participants

**Fig. 1:**
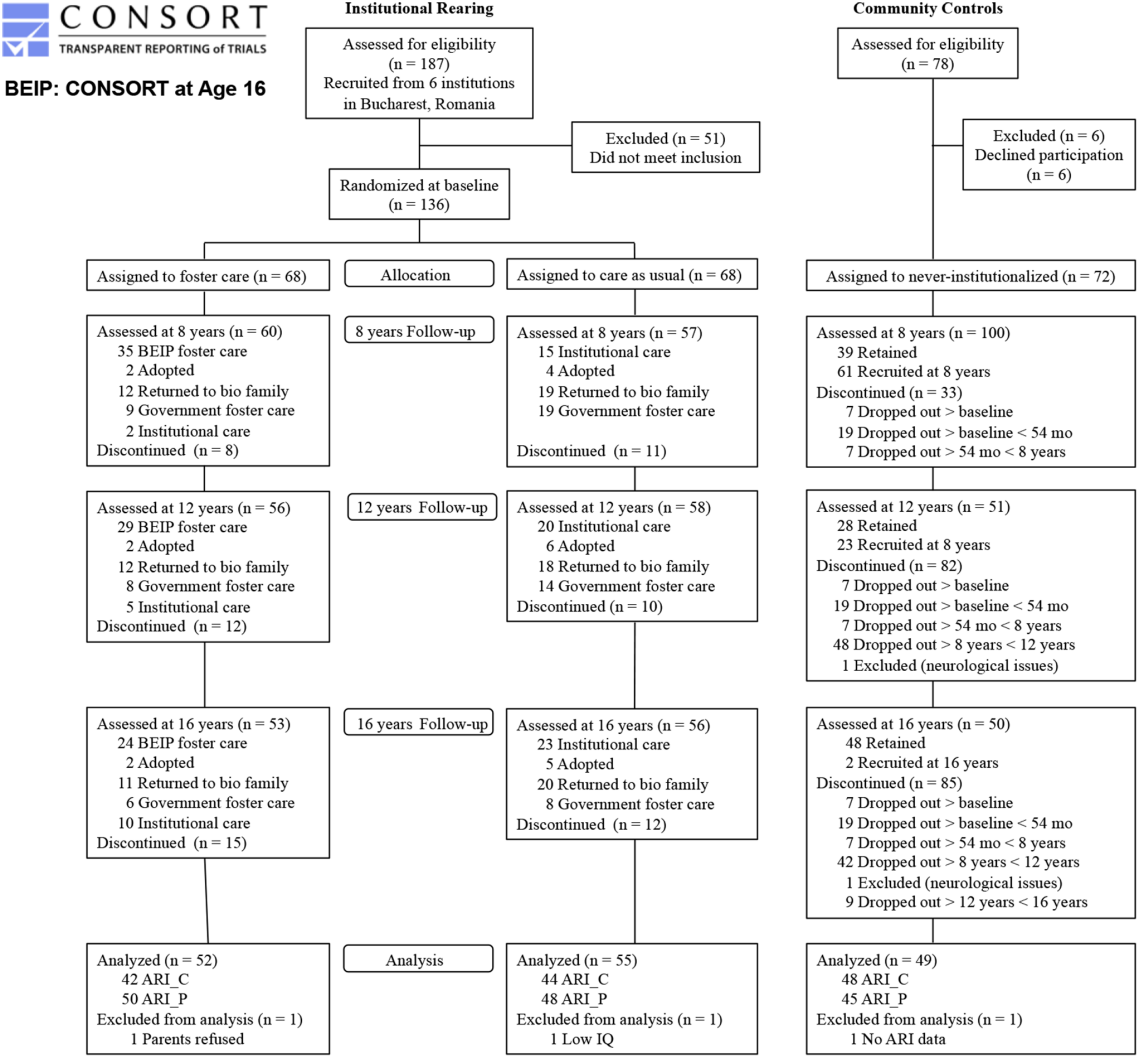
CONSORT flow diagram showing group placements over time. BEIP = Bucharest Early Intervention Project; ARI C = Child-report form of Affective Reactivity Index; ARI P = Parent-report form of Affective Reactivity Index

One hundred and thirty-six children were recruited from six sectors of Bucharest. All children less than 31 months of age in April 2001 living in institutions in Bucharest were included, with the exception of children scheduled for imminent adoption and children with serious conditions (e.g., fetal alcohol syndrome, severe cerebral palsy). Included children had no discernible genetic or neurological syndromes, nor did they show overt signs of fetal alcohol syndrome. After comprehensive assessments, the children were randomly assigned to foster care (FCG; *n* = 68) or to care as usual (CAUG; *n* = 68), meaning more prolonged institutional care (foster care was scarcely available at the time the study began). Analyses comparing the two groups at the baseline assessments indicated that there were no significant differences in age, developmental quotient, height, weight, or head circumference (King, 2022). Informed consent was obtained from each child’s legal guardian, and assent was obtained from each child at study entry. Researchers adopted a policy of noninterference during the trial, meaning that children in the CAUG could move into other placements–domestic adoptions, return to biological parents, government foster care that became more available as study progressed–as they became available, and children from both groups changed placements over time (see CONSORT diagram in Fig. 1 for the flow of participants over time). Comparisons between the FCG and the CAUG reflect their original placement assignment (i.e., intent to treat), regardless of current placement (unless otherwise stated). The FCG and CAUG are referred to as the ever-institutionalized group (EIG), while a never-institutionalized group (NIG) of 72 participants were recruited from a pediatric community health center in Bucharest. For other details of the study sample, see Zeanah and colleagues (2003). The ethics of the study have been widely discussed (Millum & Emanuel, 2007; Miller, 2009; Rid 2012; Zeanah et al., 2006, 2012).

The sample for the current report consists of 156 children (55 in CAUG, 52 in FCG, and 49 in NIG) (Zeanah et al., 2003). Participants (52% female, 48% male) were assessed at a mean age of 16.64 years (SD = 0.55) as part of the BEIP. Following approvals by the institutional review boards of the three principal investigators’ universities (University of Minnesota, University of Maryland, and Tulane University; later also Boston Children’s Hospital/Harvard Medical School and the Bucharest University Ethics committee) and by the local Commissions on Child Protection in Bucharest, the study commenced in collaboration with the Institute of Maternal and Child Health of the Romanian Ministry of Health. A data safety monitoring board in Bucharest reviewed the assessments for the current follow-up, which were also approved by the Ethics Committee of the University of Bucharest. Written (or verbal) informed consent was obtained from all participants at the 16-year assessment.

### Irritability

Irritability was assessed via parent- and self-report forms of the Affective Reactivity Index (Stringaris et al., 2012). This six-item scale, rated from 0 (*not true*) to 2 (*certainly true*), measures irritability over the past 6 months. Parent- and self-report forms of ARI are identical except for instruction sentences. For parent-report assessments, the informant varied depending on the child’s living situation: for participants in family placements, either a parent or foster parent served as the informant, whereas for those in group-based care, the caregiver who knew the child best provided the parent report. In the current sample, the internal consistencies of the parent- and self-report forms of ARI were α = 0.90 and α = 0.81, respectively. An ARI composite score was created from the parent- and self-report forms of ARI: using the mean value if both were available or the single report if only one was present. Of the 156 participants with ARI data, 121 (78%) had both parent and child reports, 13 (8%) had child reports only, and 22 (14%) had parent reports only.

### Internalizing and Externalizing Symptoms

The present study used the MacArthur Health and Behavior Questionnaire (HBQ; Essex et al., 2002) to evaluate internalizing and externalizing symptoms in children. The HBQ is a reliable and valid parent- and teacher-report instrument for assessing multiple dimensions of health and dysfunction in children. At the age of 16, both caregivers and teachers provided responses to the HBQ items, utilizing a 3-point Likert-type scale. This scale ranges from 0 (*never or not true*) to 2 (*often or very true*). The assessment of externalizing symptoms includes three subscales: oppositional defiant, conduct problems, and overt aggression. For internalizing symptoms, the parent-reported HBQ consists of subscales for depression, separation anxiety, and over-anxiousness, while the teacher-reported HBQ version includes only the depression and over-anxiousness subscales. We computed a composite score by integrating data from both the parent- and teacher-reports, using the same approach as used in the ARI. These composite scores for internalizing and externalizing symptoms were then utilized to assess their relationship with irritability. The HBQ has previously shown strong internal consistency, with Cronbach’s alphas ranging from 0.92 to 0.94 for externalizing subscales and from 0.72 to 0.87 for internalizing subscales within the BEIP sample (Tang et al., 2019).

### Baseline Irritability Assessment

The Infant-Toddler Social and Emotional Assessment (ITSEA) is a 166-item caregiver report questionnaire used to assess problem behaviors and competencies in children greater than 12 months of age (Carter et al., 2003). In the present study, negative emotionality was selected to evaluate potential baseline differences in irritability, given that this may most closely capture the early manifestations of irritability (Leibenluft & Stoddard, 2013). For the EIG, the ITSEA was administered at baseline to an institutional caregiver identified as the child’s preferred caregiver by staff consensus or, in the absence of a preferred caregiver, by one who regularly interacted with the child. The ITSEA subscales and domains have demonstrated good internal consistency, with a Cronbach’s alpha reported as 0.84 for the negative emotionality subscale (Carter et al., 2003).

### Statistical Analysis

Analyses examining irritability were conducted in R version 4.2.1 (R Core Team, 2013). First, we assessed the associations between ARI and internalizing and externalizing symptoms derived from HBQ in order to explore its overlap with these dimensions and confirm that within this sample the assessment of irritability is correlated with both internalizing and externalizing signs of psychopathology. Then, we compared ARI composite scores of ever-institutionalized and never-institutionalized individuals to examine whether institutionalization experiences are associated with differences in irritability, regardless of the foster care intervention. Next, we assessed the foster care intervention’s causal effect by comparing ARI scores between FCG and CAUG. Consistent with prior BEIP studies (see Zeanah et al., 2009), we used an intent-to-treat (ITT) analysis approach, in which participants were analyzed based on the group assigned to at the study baseline. Although many children’s living situations changed over time, the ITT analytical approach represents a conservative estimate of the intervention’s effect. To further strengthen tests of the causal impact of the intervention, we examined whether the ITT groups differed in terms of baseline negative emotionality while controlling for age at baseline assessment. Then, we further included baseline negative emotionality as a covariate in the analyses of irritability at 16 years of age, which tests for whether the RCT was associated with differences in irritability even after accounting for baseline individual differences in negative emotionality. Lastly, we examined whether additional factors (i.e., stability of placement in foster care through age 16, the percentage of time from birth to age 16 in institutional care, age of placement into foster care, and the status of living with a family through age 16) were associated with ARI composite scores at age 16.

## Results

### Associations Between Irritability and Internalizing and Externalizing Symptoms

In order to examine whether irritability levels related to broader dimensions of psychopathology, we first examined correlations. As expected, within all of the participants in our sample, irritability scores were significantly correlated with both internalizing (*r* =.27, 95%CI[0.12, 0.42], *p* <.001) and externalizing (*r* =.56, 95CI%[0.44, 0.66], *p* <.001) symptoms, such that higher levels of irritability were associated with greater severity of both internalizing and externalizing symptoms. The same pattern was found when restricting only to the ever-institutionalized children: internalizing (*r* =.24, 95%CI[0.04, 0.41], *p* =.018) and externalizing (*r* =.54, 95CI%[0.38, 0.66], *p* <.001) symptoms.

### Ever-Institutionalized with Never-Institutionalized Children

**Fig. 2:**
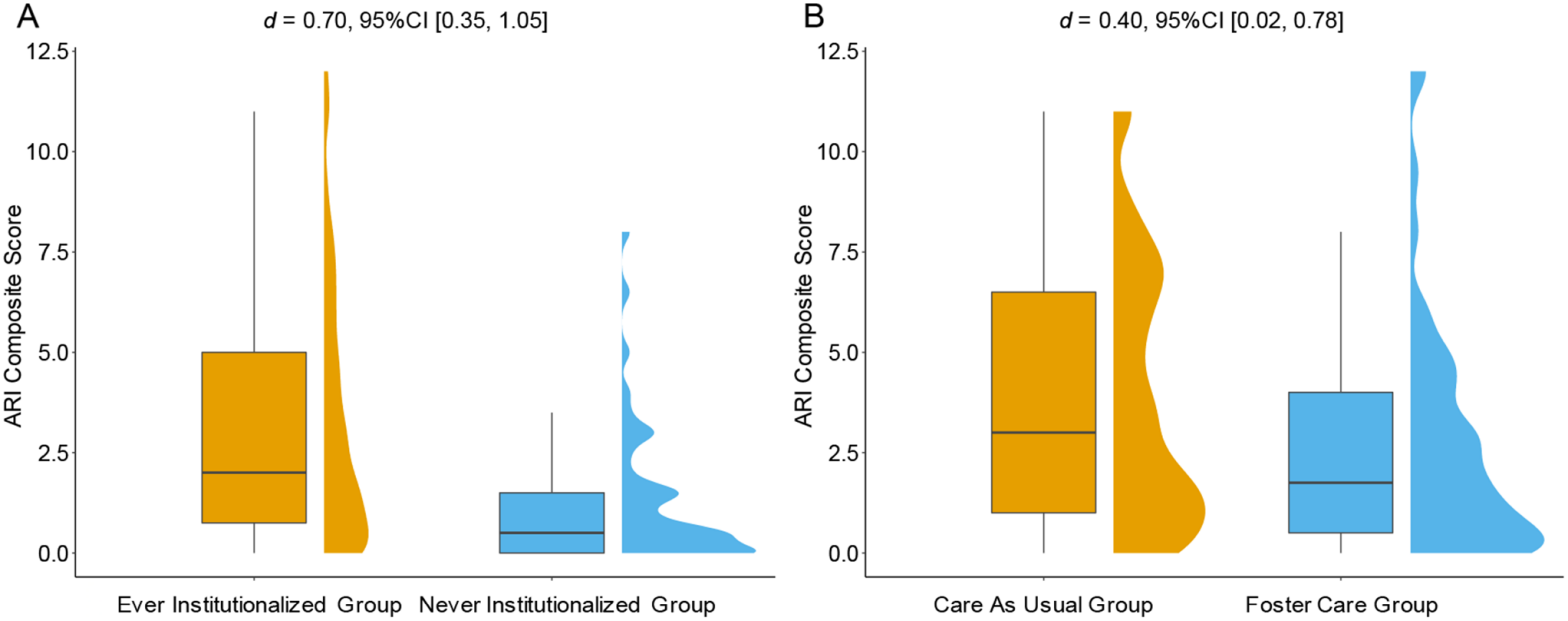
Affective Reactivity Index (ARI) composite scores at age 16 y as a function of groups. (**A**) ARI composite scores for those with a history of institutional care (ever-institutionalized) were significantly higher than those never-institutionalized. (**B**) ARI composite scores were significantly higher in the care-as-usual group (CAUG) than in the foster care group (FCG)

We then compared those with a history of institutional care to those never institutionalized. History of institutional care was associated with irritability at age 16 years (*t*(147.65) = 4.95, *p <*.001, *d* = 0.70, 95%CI [0.35, 1.05]), such that participants with any history of institutionalization (the CAUG and FCG considered together) exhibited higher levels of irritability compared to never institutionalized community comparison participants (see Fig. 2A). On average, the ARI composite score for those with a history of institutional care (*M* = 3.22, *SD* = 3.11, *n* = 107) was 1.93 points higher than those in NIG (*M* = 1.29, *SD* = 1.75, *n* = 49).

### Effects of the Intervention on Irritability

We compared irritability scores between those randomly assigned to FCG and the CAUG using an ITT analysis. Children in CAUG had significantly higher levels of irritability compared to FCG children (*t*(104.77) = 2.08, *p =*.040, *d* = 0.40, 95%CI [0.02, 0.78], see Fig. 2B). On average, irritability scores were significantly higher in the CAUG (*M* = 3.82, *SD* = 3.21, *n* = 55) than in the FCG (*M* = 2.59, *SD* = 2.90, *n* = 52).

We then took two steps to test the robustness of the RCT on irritability. First, we assessed baseline differences in the negative emotionality subscale of the ITSEA among the original 136 participants randomized at baseline. There were no statistically significant differences in negative emotionality (*β* = 0.07, 95%CI [−0.30, 0.43], *p =*.723), covarying for age at baseline, indicating that the randomization led to roughly equivalent groups in terms of levels of negative emotionality. Similarly, for the subset of participants included in the age 16 irritability analyses, we also observed no statistically significant baseline differences in negative emotionality (*β* = 0.18, 95%CI [-0.21, 0.58], *p =*.362). Second, we examined the original results from the ITT analysis on irritability at age 16 years after accounting for baseline negative emotionality. The ITT grouping were statistically significantly associated with irritability at age 16 years (*β* = 0.53, 95%CI [0.11, 0.95], *p* =.015) even after including baseline negative emotionality as a covariate.

For completeness, we also assessed baseline differences in the negative emotionality subscale of the ITSEA among ever vs. never institutionalized children. Covarying for age at baseline, there were statistically significant differences in negative emotionality (*β*=-0.52, 95%CI [−0.84, −0.21], *p =*.001), such that children residing in institutional care exhibited greater negative emotionality compared to those never institutionalized.

### Exploratory Analyses

**Fig. 3:**
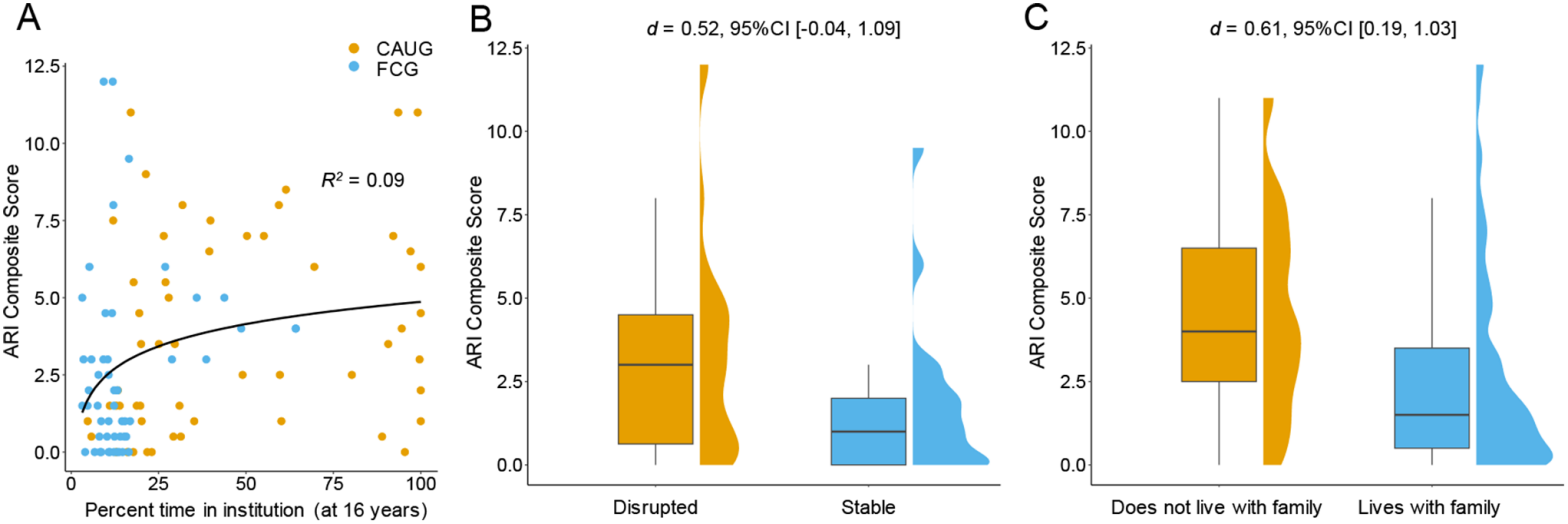
Exploratory Analyses. (**A**) There was a significant association of percent time in institutional care through age 16 with Affective Reactivity Index (ARI) composite scores. Note that care-as-usual group (CAUG) was colored in orange and the foster care group (FCG) was colored in blue. (**B**) There was a marginally significant difference in ARI composite scores between children who had stable placement in foster care and those who experienced a placement disruption by age 16 years. (**C**) Residing with a family was significantly associated with ARI composite scores

We conducted additional analyses within the ever-institutionalized group. Percent time in institutional care through age 16 years was examined in relation to irritability using both linear and linear-log models. Results indicated that the linear-log model explained greater variance in irritability than did the linear model (*R*^*2*^_linear − log model_=0.09 vs. *R*^*2*^_linear model_=0.08). According to the linear-log model, there was a significant association of percent time in institutional care through age 16 with irritability (*β* = 0.30, 95%CI [0.12, 0.49], *p* =.001, *R*^*2*^ = 0.09). As shown in Fig. 3A, those who have spent longer time in institutional care tended to have higher levels of irritability. The pattern observed was non-linear, where the association between greater time in institutional care and irritability reached a plateau as the duration of institutional care approached 100%. This suggests that, while extended periods in institutional care are generally associated with heightened irritability, there seems to be a saturation point at which additional time in care no longer corresponds to further increases in irritability.

In the foster care group, there was no association between age of placement into foster care and irritability (*β*=-0.11, 95%CI [0.42, 0.21], *p* =.49, *R*^*2*^ = 0.01). Moreover, although the difference in irritability scores between FCG children with stable foster care placements and those who experienced placement disruptions by age 16 was not statistically significant, the effect size was moderate (t(48.90) = 1.95, *p* =.057, d = 0.52, 95%CI [-0.04, 1.09], see Fig. 3B). The mean irritability score for those with a stable placement in foster care was not significantly lower (*M* = 1.76, *SD* = 2.29, *n* = 21) than those with disrupted placements (*M* = 3.25, *SD* = 3.15, *n* = 30). Lastly, we tested whether *current* placement at the time of assessment (i.e., whether or not the child was currently residing in a family, regardless of group status or stability of placement) was associated with irritability. Residing in a family was strongly associated with irritability (*t*(62.93) = 2.93, *p =*.004, *d* = 0.61, 95%CI [0.19, 1.03], see Fig. 3C), such that children who lived in a family setting (e.g., foster family, adoptive family, or reunited with their biological family) had lower levels of irritability (*M* = 2.66, *SD* = 3.03, *n* = 74) than those who were not residing with a family (*M* = 4.48, *SD* = 2.96, *n* = 33).

## Discussion

In the current study we sought to investigate the effect of a high-quality foster care intervention versus care as usual (typically prolonged institutional rearing) on irritability among adolescents. By age 16, individuals with institutional care histories demonstrated significantly elevated irritability levels relative to never-institutionalized community comparison adolescents. More importantly, among those who spent their early lives in institutional care, those randomized to foster care exhibited reduced irritability compared to those randomized to the care-as-usual condition, who often experienced prolonged institutional rearing. Furthermore, individuals with extended exposure to institutional care, and those not living in a family setting at age 16, exhibited heightened irritability levels. These findings provide experimental evidence in line with the hypothesis that the early caregiving environment affects irritability observed among adolescents who experience severe early psychosocial deprivation.

The links we observed between early caregiving environments and subsequent irritability are consistent with existing literature. First, our comparison between ever-institutionalized and never-institutionalized children suggests a significant correlation between exposure to early deprivation and heightened irritability in adolescents. This observation strengthens the existing evidence linking adverse early environments to increased irritability (Bielas et al., 2016; Oliver, 2015; Pagliaccio et al., 2018; Ravi et al., 2022; Yu et al., 2023). Additionally, our analyses revealed baseline differences in negative emotionality as a function of institutional care group, such that higher levels were found among those residing in institutional care at the start of the study compared to children who were never institutionalized. While these groups may differ in a number of ways outside of institutional care history, these group differences reflect the potential for heightened irritability related to institutional care exposure. More importantly, we then show that, among institutionalized children, those randomized to high-quality foster care displayed lower irritability levels in adolescence than those assigned to the care-as-usual group. This provides support for a causal effect of the intervention on irritability levels in adolescence. Notably, there were no differences in baseline negative emotionality, and the effect of foster care on irritability remained statistically significant even after covarying for this baseline measure. These analyses further strengthen the inferences that can be made about how foster care intervention relates to lower levels of irritability in adolescence among youth exposed to early institutionalization. The RCT design allows us to examine whether group assignment in early life was associated with levels of irritability in adolescence. However, given that children either reside in institution or family-based care, it is both true to say that the family placements that occur in foster care are associated with lower levels of irritability and that continued institutional care exposure is associated with higher levels of irritability. In other words, we are unable to divorce the effects of family placements from continued institutional care. In regards to the key differences between these placements, we have found that higher-quality caregiving interactions are the likely active ingredient, or mechanism of action, for the foster care intervention (e.g., Humphreys et al., 2022). Indeed, institutions are psychosocially depriving settings characterized by low-quality caregiving interactions. The removal from those settings and exposing children to the type of responsive care that occurs within family settings was the primary goal of the intervention. Congruent with this possibility, RCT for preschool-aged children that focused on enhancing caregiver responsiveness, positive behavior reinforcement, and improving relationships, was associated with lower levels of irritability (Smith et al., 2019). Our current finding further expands efficacy timeline of intervention, demonstrating the enduring effectiveness of early caregiving interventions well into late adolescence, and thus highlights the promise of early childhood programs focused on enhancing the caregiving environment. Please note that due to the noninterference policy, children from both the FCG and CAUG groups experienced placement changes over time. Thus, the current ITT approach may reflect an underestimation of the intervention’s true effect. Further, in line with the findings demonstrated by Buzzell et al. (2020) that foster care resulted in lower scores in general psychopathology (the “P factor”), our study highlights the association between foster care, as an alternative to institutional care, and the more clearly observable and measurable construct of irritability. Irritability is also of high transdiagnostic relevance, and more importantly, irritability, in contrast to other transdiagnostic factors that might be less apparent and more complex to identify, presents particularly early in development. Therefore, irritability offers substantial value for the early intervention strategies, gaining considerable research and clinical interest.

Additionally, we found that irritability at age 16 was associated with several other factors, including duration of institutional care, stability of foster care placements, and whether the individual lived in a family setting at age 16. Each of these markers of the early caregiving environment is potentially meaningful, though their inter-correlation makes it difficult to determine whether a specific aspect (e.g., reducing exposure to institutional care; experiencing stability in the family placement) is the primary driver of the foster care effect. Unlike the intent-to-treat analyses, these environmental characteristics were not randomly assigned, and causality cannot be inferred. It is possible that individuals with greater irritability, independent of randomization, were less likely to be placed or remain in a family, resulting in prolonged institutional care exposure and unstable placements. Interestingly, we observed relatively larger effect sizes for the percent time spent in institutional care and whether the child was currently residing with a family (Cohen’s *d* = 0.52 and 0.61 respectively) compared to the intent-to-treat effect size (*d* = 0.40). The relatively larger effect sizes may be a result of a combination of cumulative effects of the intent-to-treat effect, as well as the potential for individual predispositions in child irritability eliciting different environments. Alternatively, these larger effects may be observed because the intent-to-treat analysis, which focuses solely on the randomized aspect of the intervention, tends to be inherently more conservative in its estimates given that children are analyzed based on their initial condition rather than based on what actually occurred in their caregiving environment.

Several limitations merit consideration. First, the sample size for this study is relatively small, which may impact the precision of statistical estimates. This limitation also restricts our ability to explore more nuanced research questions important to adolescents, such as potential sex differences as a function of institutional care history or ITT group. While our exploratory analyses indicated no significant sex differences in ARI scores across the FCG, CAUG, and NIG, nor any interaction effects by ITT or between EIG and NIG groups, these findings should be interpreted with caution due to the small cell sizes. Further, while we demonstrated no baseline differences in negative emotionality—the subscale most closely aligned with irritability—our study lacked a direct assessment of irritability prior to age 16. Third, it is important to note that irritability is a transdiagnostic feature, commonly presenting in both internalizing and externalizing disorders. Therefore, the findings of our study, while focused on irritability, may not be exclusively attributable to irritability symptoms. This overlap suggests that the observed results could also be reflective of broader psychopathological factors. Fourth, we created a composite score from the parent- and self-report forms of ARI to maximize the sample size, but it can potentially introduce informant inconsistency. Including additional informants, such as teachers and peers, could enhance the reports’ validity. Lastly, it’s worth noting that the ARI is a dimensional, rather than categorical, evaluation of irritability, and does not include a threshold that signifies whether the irritability is functionally impairing. Indeed, groups differ in their approaches to measure irritability (Toohey & DiGiuseppe, 2017). For example, irritability can be measured with distinct dimensions linked to different psychopathological outcomes (Cardinale et al., 2021; Hawes et al., 2020). In contrast, the ARI focuses on capturing the unidimensional aspect, specifically the expression of anger (e.g., “stays angry for a long time”, “is angry most of the time”, “gets angry frequently”) and might align well with our study’s context, being able to capture variations in irritability across a wide range of severity rather than focusing specific dimensions of behavior or emotion. Furthermore, the ARI has demonstrated good reliability and validity in both parent- and self-report formats (Stringaris et al., 2012) and offers the advantages of brevity and ease of implementation relative to time-consuming assessments such as the structured clinical interview.

In conclusion, the present investigation advances our understanding of the relations between the early caregiving environment and irritability, emphasizing the long-lasting consequences of an improved early caregiving environment on irritability among adolescents. From a policy perspective, the present study highlights the enduring benefits of family-based care over institutional care. Along with other evidence from the Bucharest Early Intervention Project (King et al., 2023; Nelson et al., 2014), policy and resources should be allocated to eliminate institutional care and promote family-based placement, such as extended kinship networks, adoption, and stable, high-quality foster care.

## Data Availability

The data and code that support the findings of this study are available from the corresponding author upon reasonable request.
